# Skeletal Muscle Quality Of Nonagenarians And Centenarians

**DOI:** 10.1093/geroni/igab046.3008

**Published:** 2021-12-17

**Authors:** Iva Miljkovic, Adam Sterczala, Emma Barinas-Mitchell, Ryan Cvejkus, Mary Feitosa, Bharat Thyagarajan, Mary Wojczynski, Joseph Zmuda

**Affiliations:** 1 University of Pittsburgh, Pittsburgh, Pennsylvania, United States; 2 Washington University School of Medicine in St. Louis, St. Louis, Missouri, United States; 3 University of Minnesota, Minneapolis, Minnesota, United States; 4 Washington University School of Medicine, Washington University School of Medicine, Missouri, United States

## Abstract

Skeletal muscle adipose tissue infiltration is hypothesized to lead to poorer muscle quality and function with aging. Indeed, skeletal muscle adiposity has emerged as a consistent, independent predictor of skeletal muscle strength, mobility, metabolic disorders, and survival among older adults. However, phenotypic features of skeletal muscle among the oldest-old remain poorly characterized. Herein, we evaluated the skeletal muscle characteristics of 54 nonagenarians and centenarians (mean age 98 years, range 90-110 years; 63% women) and 25 middle-aged individuals (mean age 54 years, range 40-59 years; 36% women) belonging to the Long Life Family Study (LLFS), an international, multicenter cohort of families with a clustering of longevity. Ultrasonography was used to measure echo intensity of the sternocleidomastoid muscle, which has a similar fiber type distribution to the rectus femoris. Greater echo intensity is indicative of lower muscle quality (greater adipose and fibrotic tissue). Current smoking, alcohol intake, and BMI were similar between the age groups. Nonagenarians and centenarians had lower grip strength (16.3 vs. 39 kg) and were less physically active (22.2% vs 66.7% exercised 1+ times per week) compared to younger individuals (P<0.001 for all). Mean±SE echo intensity, adjusted for gender, field center, BMI and physical activity was 52.1±1.7 among nonagenarians and centenarians compared to 44.2±2.4 among younger individuals (P=0.0098). Our preliminary findings suggest that nonagenarians and centenarians may have substantially lower skeletal muscle quality and strength compared to their younger aged counterparts. Additional research is needed to better understand the mechanisms leading to poorer muscle characteristics of the oldest-old.

